# Hypoxia and lactate influence VOC production in A549 lung cancer cells

**DOI:** 10.3389/fmolb.2023.1274298

**Published:** 2023-09-21

**Authors:** Takeshi Furuhashi, Yuki Matsumoto, Ryuga Ishii, Takehito Sugasawa, Shigenori Ota

**Affiliations:** ^1^ Anicom Specialty Medical Institute Inc., Tokyo, Japan; ^2^ Laboratory of Clinical Examination/Sports Medicine, Division of Clinical Medicine, Faculty of Medicine, University of Tsukuba, Tsukuba, Japan; ^3^ GL Science Inc., Saitama, Japan

**Keywords:** cellular VOC, cancer metabolism, lipid peroxidation, hypoxia, lactic acid signaling, lung cancer cell

## Abstract

**Introduction:** Cancer cells emit characteristic volatile organic compounds (VOCs), which are potentially generated from ROS-based lipid peroxidation of polyunsaturated fatty acids. The metabolism of such VOCs and their regulation remain to be fully investigated. In fact, the enzymes involved in the synthesis of these VOCs have not been described yet.

**Methods:** In this study, we firstly conducted *in vitro* enzyme assays and demonstrated that recombinant alcohol dehydrogenase (ADH) converted *Trans* 2-hexenal into *Trans* 2-hexenol. The latter has previously been reported as a cancer VOC. To study VOC metabolism, 14 different culture conditions were compared in view of *Trans* 2-hexenol production.

**Results and discussion:** The data indicate that hypoxia and the addition of lactate positively influenced *Trans* 2-hexenol production in A549 cancer cells. The RNAseq data suggested certain gene expressions in the VOC pathway and in lactate signaling, parallel to VOC production. This implies that hypoxia and lactate signaling with a VOC production can be characteristic for cancer *in vitro*.

## Introduction

Cancer odor is one of the current emergent topics in cancer research in view of preventive medicine and early detection of cancer by non-invasive clinical tests (Oxner et al., 2023). Cancers are known to emit odor. Accordingly, animals have been used for the chemical detection of cancer (e.g., dogs, nematodes and insects) (Amundsen et al., 2014; Lanza et al., 2021; Farnum et al., 2023). Although dogs, for example, can discern the odor compounds associated with cancer, this approach cannot yield profiles and definitive identifications. This calls for basic cancer odor studies, and volatile organic compounds (VOCs) have been identified and measured from various specimens (e.g., blood, urine, feces) with different analytical methods (e.g., GC-MS, PTR, SIFT, e-nose) since 1984 (Gouzerh et al., 2021). In addition, odor has been detected not only in the form of VOCs from specimens but also from cultured cancer cells (Filipiak et al., 2010; Furuhashi et al., 2020).

**FIGURE 1:**
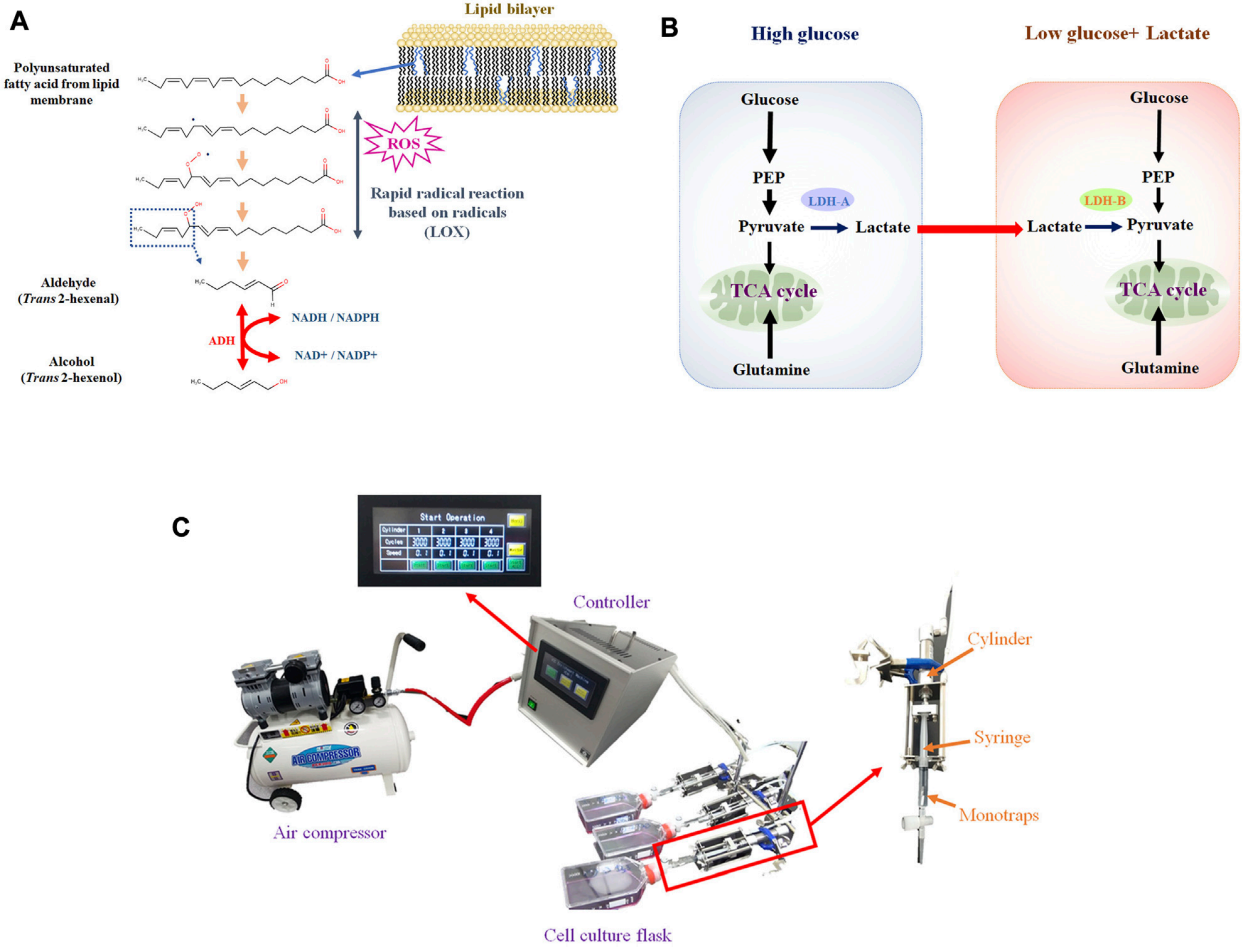
**(A)**, Hypothetical VOC production pathway originated from lipid peroxidation of membrane lipids. Aldehyde is generated from PUFA by LOX, based on radical reaction. Aldehyde can be converted into alcohol by ADH; **(B)**, Lactate shuttle between cancer cells under high glucose (glycolytic) and low glucose. Lactate can be secreted from cells under high glucose and consumed under low glucose; **(C)**, VEM-1 (VOC Enrichment Machine), automated pumping machine connected to air compressor that drives pumping. Monotraps (RG and RSC18) were put into a 2.5 mL disposable syringe (TERMO, SS-02SZ) connected to a cylinder.

Most previous studies, however, have focused exclusively on the VOCs profile, whereby the metabolism of VOC production at the cellular level has not been fully considered. Cancer VOCs would originate from lipid peroxidation of membrane lipids (Figure 1A). Accordingly, rapid cell proliferation leads to reactive oxygen species (ROS) production, and ROS cause lipid peroxidation of membrane polyunsaturated fatty acids (Medeiros, 2019; Arfin et al., 2021). Nonetheless, some key points are missing, in particular enzymatic activity data. Enzyme assays, however, can be challenging because VOCs are volatile. As we previously reported, *trans* 2 hexenol could be one of the cancer VOC candidates, and alcohol dehydrogenase (ADH) would be the required enzyme for synthesis (Furuhashi et al., 2020). Another issue is the regulation of VOC metabolism by ADH. As certain environmental factors can influence both the metabolism and production of VOCs (Figure 1B) (Brooks, 2018; Kim, 2018). For instance, it can be envisaged that hypoxia can influence VOC production because lipid peroxidation requires oxygen. Enhanced glucose uptake and glycolysis, known as the Warburg effect, is characteristic of cancer cells, but no reports are available that have investigated the relation to cancer cell-specific VOC production. Glutaminolysis and lactate can be related to either ROS production or signaling. Based on these considerations, specifying such factors is crucial to better understand cancer VOC metabolism. As a first step, we therefore compared different culture conditions (i.e., hypoxia, glucose, glutamine, lactate) in cancerous and non-cancerous lung cell lines to determine important factors for VOC production.

## Materials and methods

### ADH enzyme assay *in vitro*

ADH enzyme activity was assayed using ADH1c Recombinant Human ADH1C protein (100 μg; Abcum, ab128432). *Trans* 2-hexenol as a product was derivatized and identified by GC-MS.

Substrate (5 μL 100 mM trans-2-hexenal) as substrate was added in a 2 mL Eppendorf tube with 200 μL 50 mM Tris-HCl buffer (pH 8.0 with HCl). Coenzyme, either 5 μL of 10 mM NADH or 10 mM NADPH, was added. After mixing, 2 μL ADH1c (0.5 mg/mL 50 mM Tris-HCl) was added and incubated at 30°C for 30 min. After that, 150 μL dichloromethane was added and phase separation was done by vortexing and subsequent centrifugation at 20,000 g for 2 min. 50 μL bottom dichloromethane phase was transferred into a new 2 mL Eppendorf tube. 50 μL MSTFA was added and incubated at 37°C for 30 min 1 μL was then loaded into GC-MS. Data on the negative control was obtained without substrate.

The GC column (HP-5MS), the oven temperature gradient (50°C isothermal period 5 min, the oven was programmed to rise to 150°C at a rate of 5°C min^−1^, and then rise to 330°C at a rate of 40°C min^−1^, held at 330°C for 6 min) and the GC-MS measurements were carried out using the same instruments and methods as described in a previous study (Furuhashi et al., 2018).

The peak area of a conventional 70 eV EI mode (Extractor ion source; Agilent Technologies, Santa Clara, CA, United States) extracted ion chromatogram (EIC) was determined using software for quantification (Mass Hunter Quantitative analysis B.07.01SP1; Agilent Technologies, Santa Clara, CA, United States). *m/z* 129 was used for absolute quantification with RT window 10 range between 2.5 and 500 nM and LOD (limit of detection) was around 500 pmole.

### Cell culture conditions

All cell lines used in this study—cancer cell line (A549 cells and human lung fibroblasts), non-cancer immortalized cell (HLB), and primary cells [Human bronchial epithelial cells (HBEpC)]—were same as in a previous study (Furuhashi et al., 2020).

Prior to comparing the different media, all cells were (2 × 10^6^) grown in DMEM (Dulbecco’s modified eagle medium) high-glucose culture medium containing sodium pyruvate (110 mg/L) supplemented with 10% FCS (fetal bovine serum), penicillin (100,000 units/L), streptomycin (100 mg/L), and L-glutamine (293 mg/L) with T75 cell culture flask (250 mL, 75 cm^2^) under standard conditions at 37°C in humidified atmosphere with 92.5% air/7.5%CO_2_ (used as the normoxia condition). Cell Counter model R1 (OLYMPUS, United States) was used for cell counts. The media used for the culture condition comparison are described in Supplementary Figure S1.

For glutamine deprivation, GIBCO DMEM, high glucose, no glutamine (10313021; Thermo-Fischer, United States) was used. Low-glucose medium was prepared by adding glucose (16806-25, Nacalai tesque, Kyoto, Japan) into GIBCO DMEM, no glucose (11966025; Thermo-Fischer, United States) to a final concentration of 1 g glucose/mL medium. L-Lactic acid (L0165, TCI, Tokyo, Japan) was used for the lactate addition medium. Glucose and lactate were added with filtration by using Millex-HP 0.45 μm (SLHP033RS, Millipore, United States).

Hypoxia conditions were developed by modifying a previous protocol (Itoi et al., 2012). In this study, the culture flask was placed into a gas-barrier bag (Smart bag PA 3008 (600 mm × 350 mm) (A026-F0L14, GL Sciences, Saitama, Japan)) and an oxygen absorber was added (A-07, Sugiyamagen, Tokyo, Japan). An O_2_ concentration meter (OXY-2, Sugiyamagen, Tokyo, Japan) monitored the O_2_ concentration, because around 1% O_2_ concentration had been used for hypoxic conditions in a previous study (Guo et al., 2018). The Smart bag was then put into an incubator to maintain the temperature at 37°C. The oxygen concentration was below 5% within 4 h after opening the oxygen absorber. After the O_2_ concentration fell to below 1.5%, the space between the oxygen absorber and flask was insulated to maintain the optimal O_2_ concentration during the experiment (Day0-Day3).

Cells were grown in these conditions for 72 h, up to a confluence of 50%–60% (around 1.0 × 10^6^ cells/flask). On Day 3, cells were sampled for the precursor addition experiment, for medium sugar and lactate quantification, the cellular LDH-B activity assay, and FFAs/FAMEs analysis by GC-MS and RNAseq. On Days 0, 2, and 3, samples were collected for VOC analysis.

### VOC measurement by GC-MS

VOC enrichment with monotrap by pumping and quantifying trans-2-hexenol was described previously (Furuhashi et al., 2020). The life-time of VOCs can be short because they can be re-dissolved in the medium or be decomposed/transformed by the cells. For this reason, we automated the pumping system in this study to perform rapid VOC capture and fixation.

The cell culture flask (T75) at each incubation period (Days 0 to Days 3) was firstly connected to a special penetrating vent cap. Cellular VOC was enriched by monotrap with an automated pumping machine called VEM-1 (VOC Enrichment Machine: PLC control mode, 199 mm height × 200 mm width × 330 mm length, 6 kg weight, AC100V, 50/60 Hz, 1 A) connecting to an air compressor (Prostyle PCR3010, 30L, FLOBAL, Inc., Japan). Monotraps (RG and RSC18) were put into a 2.5 mL disposable syringe (TERMO, SS-02SZ). The syringe was connected to a cylinder and pumping was conducted 3,000 times in 10 min (Figure 1C). After pumping, monotraps were transferred to a thermal desorption machine (Handy-TD, GL Sciences) and subsequently measured by GC-MS. Each measurement was biologically replicated 3 times.

GC-MS measurements were carried out on a single quadrupole mass spectrometer (5977B-MSD; Agilent Technologies, Santa Clara, CA, United States) equipped with 7890BGC (Agilent Technologies, Santa Clara, CA, United States) and Handy-TD265 (GL science, Iruma city, Japan). The conditions (liner, GC column setting, oven temperature gradient, GC-MS ion source and transfer line, scan range) were the same as described in a previous study (Furuhashi et al., 2020). For VOC (i.e., trans-2-hexenol) quantification, *m/z* 82 was used for absolute quantification with RT window 10, and a calibration curve (5 nM–500 nM) was prepared using a reference compound (trans-2-hexenol, SIGMA 132667) with using software (Mass Hunter Quantitative analysis B.07.01SP1; Agilent Technologies, Santa Clara, CA, United States). LOD was around 2.5 nmole. For normalization, C17:0 FAME standard was measured between each batch analysis.

### Precursor (*Trans* 2-hexenal) addition

A549 cells were (2 × 10^6^) grown in DMEM high-glucose culture medium under standard aerobic conditions with or without 1nmole trans-2-hexenal addition to the medium.

Cellular VOC at Day3 was enriched by monotrap by VEM-1 and measured by GC-MS as in the VOC measurement protocol above.

### Glucose consumption by phenol-sulfuric acid assay

Glucose consumption was estimated based on the decrease of glucose (i.e., neutral sugars quantified by Phenol-Sulfuric Acid Assay whose protocol was the same as in a previous study (Furuhashi et al., 2020). At Day 0 and Day 3, 5 μL culture medium was collected and mixed with 195 μL deionized water. Thereafter, 80% 5 μL phenol solution and 500 μL of concentrated sulfuric acid was added by titration (on ice). After mixing by inverting the tube and incubating for 25 min at room temperature, absorbency at 490 nm was measured using Enspire (Perkin-Elmer, United States). Each measurement was biologically replicated 3 times. Quantification involved using a linear calibration curve with glucose standard solution (0.5–100 μg).

### Medium lactate assay

Lactate production and secretion outside the cell was estimated from the medium lactate concentration by using a Lactic acid Assay Kit (10139084035, R-Biopharm AG, Germany). At Day 3, 1 μL culture medium was collected and mixed with 99 μL deionized water. Then, 100 μL glycylglycine buffer (440 mg/30 mL, pH 10), 20 μL NAD (nicotinamide adenine dinucleotide) solution (210 mg/6 mL) and 2 μL glutamate-pyruvate transaminase suspension (1,100 U) was added. After incubation at room temperature for 5 min, absorbency at 340 nm was measured by Enspire before (A1) and after (A2) addition of 2 μL lactate dehydrogenase solution (3,800 U). The lactate concentration was calculated as A2-A1. Each measurement was biologically replicated 3 times. A linear calibration curve with lactate standard solution (10–2,000 ng) was used for quantification.

### LDH-B enzyme activity

The Lactate Dehydrogenase Assay Kit (Colorimetric) (500 tests) (Abcam: ab102526) was used to quantify cellular LDH-B activity. 2 × 10^6^ Cells (HBEpC, HLB, and A549) in star T75 flask cells at Day 3 were used, and cells were detached from the flask with a scraper. The cell suspension was washed with PBS (phosphate buffered saline) and prepared according to the manufacturer’s protocol. Absorbency at 450 nm was measured by Enspire at time 0 for the NADH concentration, 10 min after incubation at 37°C for LDH-B activity. A linear calibration curve with NADH standard solution (0.1–50 nmole) was used for quantification. NADH was calculated as nmole/cell. LDH-B activity was calculated as ΔNADH nmole/min/cell (ΔNADH between 0 and 10 min).

### RNAseq

For RNA extraction, 2 × 10^6^ cells (HBEpC, HLB, and A549) in star T75 flask at Day 3 were used. Culture conditions were compared between aerobic high glucose and hypoxia low glucose with and without lactate (0, 12.5 μM, 125 μM, and 1.25 mM). After trypsin treatment, cells were washed with PBS two times (phosphate buffered saline). Total RNA was extracted from cells using RNeasy plus mini kit 250 (QIAGEN, 74136) according to the manufacturer’s protocol.

### A complementary DNA (cDNA) library was constructed using NEBNext

Poly (A) mRNA Magnetic Isolation Module (Cat#E7490) and NEBNext Ultra II RNA Library Prep Kit for Illumina (Cat#E7770L), according to the manufacturer’s protocol. The cDNA library was sequenced on an Illumina NextSeq 500 (Illumina, San Diego, United States) using the NextSeq 500/550 High Output Kit v2.0 (Illumina). The read length was 36 bp paired-end.

Low-quality sequenced reads were trimmed using trim galore (0.6.6) with the default settings. Moreover, transcripts per million (TPM) were used for RNA quantification as well as subsequent statistical analysis. The reference cDNA datasets were GRCh38 based annotations deposited in the Ensembl genome browser (release 106). Gene expression was quantified using the Kallisto/Sleuth pipeline. Briefly, Kallisto (v0.46.0) is a pseudoalignment-based method used to quantify RNA abundance at the transcriptional level (PMID: 27043002). The TPM was calculated using the quant option implemented in Kallisto. Thereafter, the downstream differential gene expression analysis was analyzed by Sleuth (v0.30.0) using the TPM data (PMID: 28581496). All statistical tests were corrected by the Benjamini–Hochberg method, and the statistical significance level was set as false discovery rate (FDR) <0.1 in the Wald test.

### FFA and FAME analysis by GC-MS

1 mL of MCW solvent (methanol/chloroform, 5:2, v/v) was added to the Day 3 cell sample and homogenized mechanically (Precellys Evolution; Bertin Technologies, France) at 6,000 rpm for 20 s twice with a 30 s interval. Phase separation was conducted by adding 500 µL of water and 400 µL of chloroform to the supernatant. An upper polar phase was removed by centrifuging at 21,000 × ɡ for 3 min. The remaining inter-phase and the bottom apolar phase were desiccated using a Centrifuge concentrator (CC-105) (TOMY, Japan).

Only the bound form of fatty acids is converted (e.g., triglycerides and phospholipid) into FAMEs by methyl esterification, whereas free fatty acids (FFAs) and steroids contained in the original sample were silylated during the subsequent MSTFA treatment (Furuhashi et al., 2016). The dried apolar (with inter-phase) pellet was incubated with 500 µL of 0.5 M sodium methoxide in methanol for 90 min at 60°C. After cooling down to room temperature, 1 mL of 1% acetic acid and 400 µL chloroform was added to stop the reaction. Phase separation was conducted by centrifugation at 21,000 × ɡ for 3 min. After the upper polar phase was removed, 1 mL of ultrapure water was added. An upper polar phase was removed again by centrifugation at 21,000 × ɡ for 3 min. The remaining inter-phase and the bottom apolar phase were desiccated using a centrifuge concentrator. The dried pellet was incubated with 10 µL of pyridine solution for 30 min at room temperature and 40 µL MSTFA was added and incubated for 30 min at 37°C. The 1 µL of solution was injected into the GC-MS. The retention time was compared to that of authentic alkane standard compounds according to a previous study (Furuhashi et al., 2020). Measurements were performed in a batch.

GC column (HP-5MS), and GC-MS measurements were carried out using the same instrument and method as in a previous study (Furuhashi et al., 2018), except the oven temperature gradient (70°C isothermic period 4 min, the oven was programmed to rise to 330°C at a rate of 8°C min^−1^, held at 330°C for 6 min). The peak area of a conventional 70 eV EI mode (Extractor ion source; Agilent Technologies, Santa Clara, CA, United States) extracted ion chromatogram (EIC) was determined using software for relative quantification based on peak area calculation (Mass Hunter Quantitative analysis B.07.01SP1; Agilent Technologies, Santa Clara, CA, United States). The chosen retention time and *m/z* for FFAs and FAMEs quantification with RT window 10 as parameter are described in Supplementary Figure S2.

## Results and discussion

### Trans 2-hexenol conversion by ADH *in vitro*

**FIGURE 2:**
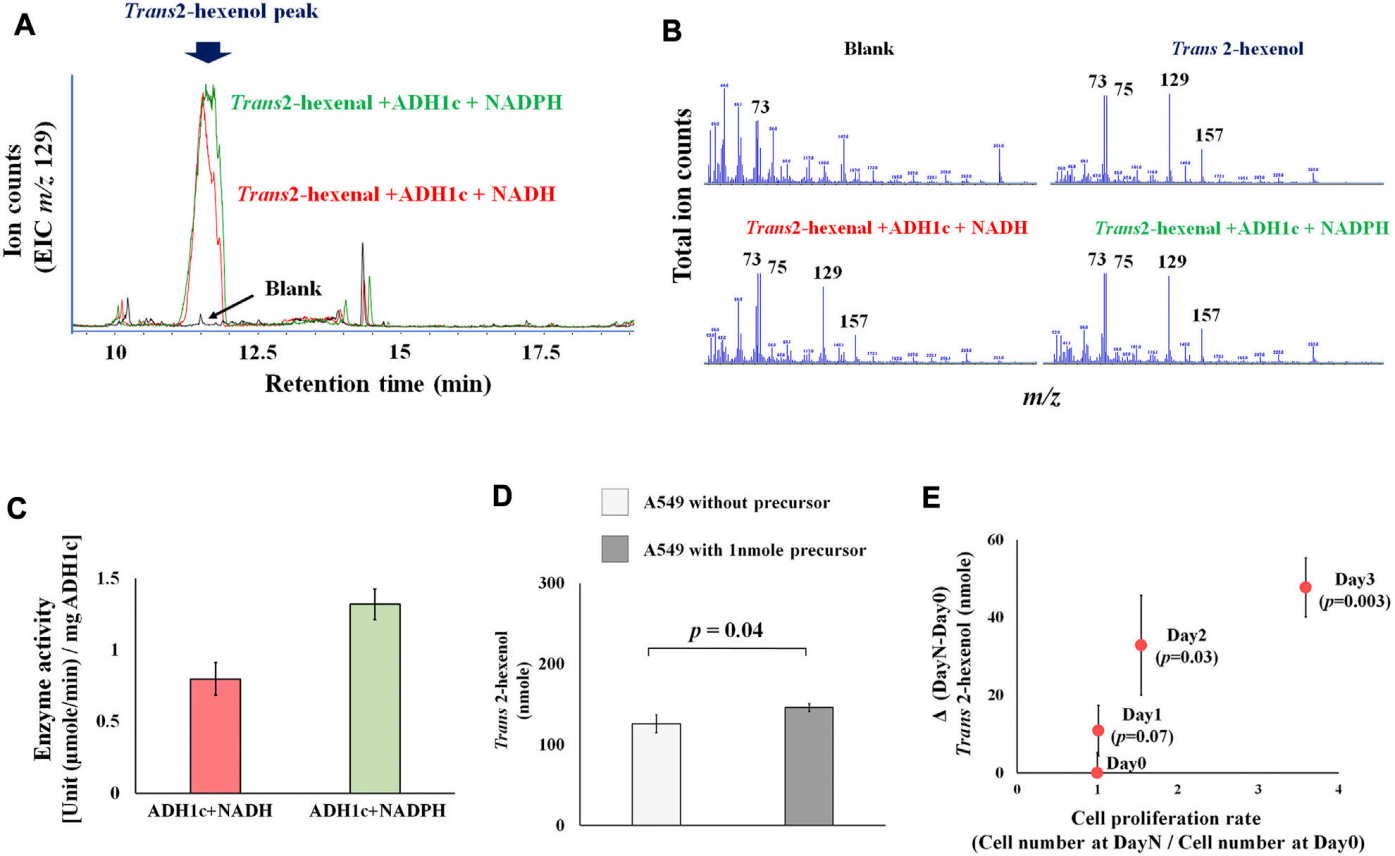
**(A)**, Extracted ion chromatogram (m/z 129) in ADH1 enzymatic assay. Peak indicates trans-2-hexanol TMS derivatives. Blank is negative control without substrate (trans-2-hexenal); **(B)**, MS spectrum of blank, trans-2-hexenol standard, trans-2-hexenol synthesized *in vitro* with NADH or NADPH. MS spectrum of generated trans-2-hexenol by ADH1 *in vitro* showed an MS spectrum identical with that of reference compounds, as characteristic fragments (m/z 73, 75, 129, 157) were common; **(C)**, ADH1 enzyme unit to catalyze trans-2-hexenal into trans-2-hexenol with NADH and NADPH; **(D)**, Precursor addition test. A549 was cultured in high glucose DMEM medium under normal aerobic conditions with or without addition of 1nmole trans-2-hexenal. Y axis is trans-2-hexenol nmole at Day 3, and the *p*-value between them was 0.04; **(E)**, Time course experiments of A549 trans-2-hexenol production. X axis is cell proliferation rate [Cell number (Day X/Day 0)] and Y axis is ΔVOC [trans-2-hexenol nmole (DayX-Day0)]. Day X is either Day 1, Day 2 or Day 3, and the *p*-values were 0.07, 0.03, and 0.003, respectively.

Previous studies concentrated on human and rat enzyme activity of ADH (class I-III), showing that various aldehydes and ketones (including 2-hexenal and cyclohexanone) can be converted into alcohol (Deetz et al., 1984; Boleda et al., 1993). These results, however, are based on the absorbance change of NADH, without any product identification. This is problematic because of enzyme promiscuity (i.e., substrates, catalytic, conditional) (Piedrafita et al., 2015), and ADH can catalyze various substrates. This makes the actual identification of products a priority. We therefore focused on identifying actual products, and successfully detected the enzyme activity of ADH, which converts the precursor *Trans* 2-hexenal into *Trans* 2-hexenol as a product. A *Trans* 2-hexenol-TMS derivative, generated *in vitro*, appeared in the GC chromatogram (Figure 2A). Importantly its MS spectrum was identical with reference compound (Figure 2B). The data showed that enzyme activity at 30°C (pH 8.0) [U (µmole/min)/mg ADH1c] was 0.8 (±0.1) with NADH and 1.3 (±0.1) with NADPH (Figure 2C). Enzyme activity was shown with both NADP and NADPH.

Regarding the precursor (*Trans* 2-hexenal), an addition experiment showed an increase of *Trans* 2-hexenol in the A549 cell culture (Figure 2D), pointing to a conversion from *Trans* 2-hexenal into *Trans* 2-hexenol in these cells.

Our data are consistent with previous studies stating that ADH can catalyze a wide range of substrates and plays a multifunctional role in cells. ROS can be generated during rapid proliferation, leading to lipid peroxidation. The lipid peroxidation process includes non-enzymatic radical reactions, and it can generate various products including harmful aldehydes. Cells need to cope with various types of chemicals flexibly, making multifunctional enzymes (i.e., ADH) important.

#### Trans 2-hexenol production under different cultural conditions

Comparing various culture conditions would help decipher the metabolic regulation of VOC production. Firstly, the time course of *Trans* 2-hexenol in A549 cell culture was determined to find the optimal sampling points (Figure 2E). An increase of *Trans* 2-hexenol became statistically significant (compared to Day 0) after 2 days incubation: 32.8 (±12.8) and 47.7 (±7.6) nmole *Trans* 2-hexenol (displayed as ΔVOC) were recorded at 2 and 3 days incubation, respectively. The number of cells increased beginning 2 days after incubation, implying that the *Trans* 2-hexenol increase was concomitant with cell proliferation.

**FIGURE 3:**
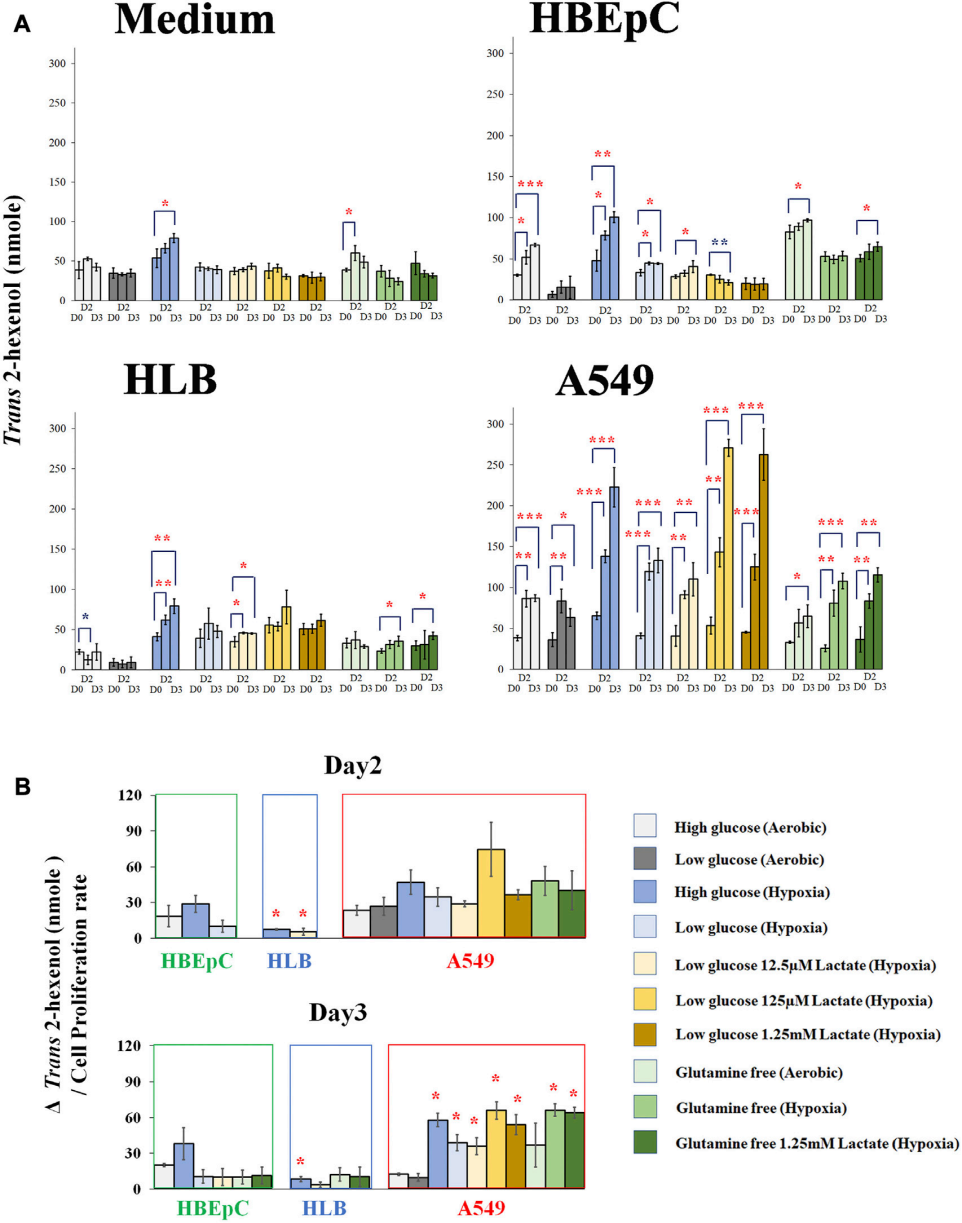
**(A)**, Quantification of *Trans* 2-hexenol nmole (Day 0, Day2 and Day 3 (negative control cell-free medium, HBEpC, HLB and A549). *, **, and ***: statistical significance (*p* < 0.05, 0.01, and 0.001, respectively). Color of * (red and blue) indicates either upregulated or downregulated. A549 at Day 2 and Day 3 showed significant VOC upregulation compared to Day 0 in almost all culture conditions; **(B)**, Δ*Trans* 2-hexenol production (nmole)/cell proliferation rate. Culture conditions showing statistical significance in Figure 3A were selected and normalized by cell proliferation rate.Δ*Trans* 2-hexenol production (nmole) is [trans-2-hexenol nmole (DayX-Day 0)]. The cell proliferation rate is [Cell number (Day X/Day 0)]. Day X is either Day 2 or Day 3. The comparison is between normal aerobic high glucose and any other culture condition in each cell line. * statistical significance based on Holm–Bonferroni method (i.e., in the case of HBEpC and HLB, *p* < 0.025 and *p* < 0.0125 at Day 2 and Day 3, respectively. As for A549, *p* < 0.0055).

We compared *Trans* 2-hexenol production under various culture conditions (e.g., hypoxia and glutamine-free) at Days 0, 2, and 3 based on the time course experiments above (Figure 3A; Supplementary Figure S3A). The medium without cells as negative control showed no clear tendency (Figure 3A). Nonetheless, some of the points showed a small increase of *Trans* 2-hexenol. The maximum increase of *Trans* 2-hexenol (i.e.,ΔVOC) in the negative control (aerobic with high glucose and no glutamine) on Day 2was 21.6 (±11.7) nmole.

As for HBEpC (normal primary human lung cell), the increase in *Trans* 2-hexenol was statistically significant under 6 different culture conditions, including normal aerobic conditions and hypoxia with low glucose and 125 µM lactate at Day 3 (Figures 3A, B). Among these, aerobic conditions with high glucose (Day 3) and hypoxia with high glucose (both Day 2 and Day 3) showed an over 20 nmole *Trans* 2-hexenol increase from Day 0 (Figure 3A).

In the case of HLB (immortalized human lung cell), no *Trans* 2-hexenol production was observed under normal aerobic conditions, but the values increased under 4 different culture conditions (Figure 3A). Among these, only hypoxia with high glucose showed an increase of *Trans* 2-hexenol above 20 nmol from Day 0.

A549 showed *Trans* 2-hexenol production in all culture conditions except aerobic with high glucose and no glutamine at Day 2 (Figure 3A). All hypoxia as well as lactate-added conditions showed significant production in A549. Hypoxia influenced *Trans* 2-hexenol production positively; in particular, it was maximized at hypoxia with low glucose and added lactate.

Culture condition comparison was also done to Day 2 and Day 3, respectively, with normalization according to cell proliferation rate (Figure 3B). The comparison conducted on Day 2 and Day 3 showed statistical significance in Figure 3A. HBEpC on both days did not show a statistically significant difference between the aerobic high glucose condition and others (e.g., hypoxia). HLB under hypoxia with high glucose (both Day 2 and Day 3) and low glucose with 12.5 µM lactate at Day 2 showed a statistically significant increase. In the case of A549, all conditions at Day 2 showed no statistically significant increase, whereas 7 different culture conditions showed such an increase at Day 3 (Figure 3B). The data suggested that the *Trans* 2-hexenol production increase under different culture conditions (e.g., hypoxia) in A549 is not only due to cell number increase but also due to possible metabolic changes.

Overall, low glucose negatively influenced VOC production for HLB and HBEpC, while a VOC increase was confirmed in A549 at both high and low glucose conditions. A positive influence of hypoxia on VOC production was common in all cell lines. This is consistent with a previous report that hypoxia enhances ROS and subsequent lipid peroxidation (Behn et al., 2007). Lactate addition did not positively affect HLB and HBEpC. Given that lactate addition was positive in A549 and that it appeared to be a unique characteristic in the A549 cancer cell line, the concentration of extracellular lactate is important. Indeed, a high concentration of lactate (e.g., 20 mM) causes toxic effects, while lower concentrations had a positive effect on cancer growth, i.e. promoted growth. As for glutamine, deprivation negatively influenced both the VOC production amount as well as cell proliferation in all cell lines. Interestingly, in A549, normalized *Trans* 2-hexenol production by cell proliferation even increased at glutamine deprivation under hypoxia, although the cell proliferation level dropped (Figure 3B; Supplementary Figure S3A). Lactate addition did not alter this influence. In summary, hypoxia and lactate appear to be key factors for A549 VOC production and metabolism, and this warrants further scrutiny.

### Glucose consumption and lactate production rate

The enhanced glucose uptake and glycolysis pathway is characteristics for proliferating cells. Regarding lactate, the hypoxia response and lactate signaling can be correlated (Park et al., 2015). Therefore, glucose consumption from the medium and lactate production were investigated to determine potential implications of glycolysis and lactate metabolism on VOC production. Glucose consumption differs between high and low glucose amounts as well as according to cell type (Supplementary Figure S4). Under low glucose conditions, there was no difference between the culture conditions in any cell type. Under high glucose conditions, glucose consumption was also not influenced by culture condition in HBEpC. In contrast, glutamine deprivation under hypoxia decreased glucose consumption in HLB and A549, suggesting that glutaminolysis affected glucose consumption of proliferating cells under high glucose and hypoxia.

Regarding lactate (i.e., lactate concentration in the medium) (Supplementary Figure S4), there was no change in HBEpC under any culture condition. In the case of HLB, lactate increased under hypoxia with high glucose and decreased under glutamine deprivation both in aerobic conditions and under hypoxia (both with and without lactate addition), compared with under the aerobic high glucose condition. Under the low glucose condition, HLB showed no difference. In A549, glutamine deprivation under hypoxia (both with and without lactate addition) in the high glucose condition decreased lactate production and secretion. Under low glucose, A549 showed a decrease of lactate in all conditions under hypoxia with lactate addition. This implies that A549 cells, under hypoxia with low glucose, appear to inhibit lactate production when the extracellular lactate concentration is high.

In the Warburg effect, the rate of glycolysis is high without affecting mitochondrial activity in most cancers, and lactate accumulation can enhance tumor progression (Vaupel et al., 2019). The rate of glycolysis and glutaminolysis is high in cancer cells under both aerobic conditions and hypoxia, and lactate would contribute to angiogenesis (Polet and Feron, 2013). Our data also inferred that the link between glycolysis and glutaminolysis is common in proliferating cells. Moreover, a decrease in glutamine leads to a decrease in glucose consumption due to a crosstalk mechanism, i.e. CtBP-SIRT4-GDH signal transduction (Wang et al., 2018).

Based on an influence of hypoxia and lactate addition onA549 VOC production, we analyzed LDH-B (Supplementary Figure S3B). LDH-B is important in lactate metabolism, converting lactate into pyruvate. There is variation in LDH (i.e., LD1-LD5) (Jafary et al., 2019), and LDHA converts pyruvate into lactate, while LDHB converts lactate into pyruvate (Marchiq and Pouysségur, 2016). Accordingly, enzymatic assays of LDH-B are perhaps more important than solely determining the gene expression level of LDH.

There was no significant change in LDH-B of HBEpC under any culture condition. Regarding LDH-B of HLB, it increased only in glutamine deprivation under aerobic conditions in HLB. In HLB, no clear correlation between LDH-B activity and change in cellular lactate production and secretion was evident. A549 LDH-B increased only under hypoxia (except under low glucose with 12.5 μM lactate addition). The decrease in lactate production and secretion under hypoxia with low glucose and lactate addition was not due to loss by lactate conversion to pyruvate by LHD-B increase.

#### Gene expression (RNAseq)

**FIGURE 4:**
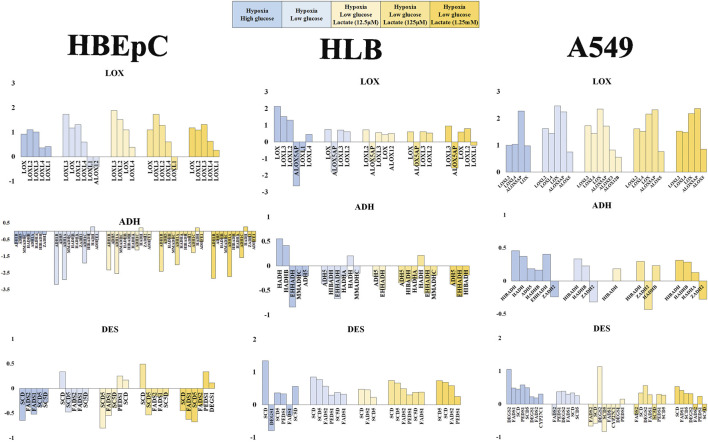
Mean fold value reflecting change in gene expression pattern based on RNAseq data (HBEpC, HLB and A549). Lipid peroxidation (VOC synthesis) pathway (DES, LOX, ADH), glucose consumption and glycolysis (SLC2A, HK), and lactate metabolism and signaling (LDH, NDGR) are three main focuses. Six different culture conditions were compared, normoxia high glucose, hypoxia high glucose, hypoxia low glucose, and hypoxia low glucose with lactate (12.5 μM, 125 μM, or 1.25 mM). Only annotated genes showing statistical significance (*q* < 0.05) were displayed with either upregulated or downregulated compared to the control, which is the normoxia high glucose condition (Supplementary Material). ADH, alcohol dehydrogenase and related oxide reductase; DES, desaturase; LOX, lipoxygenases related enzyme.

Based on the influence of hypoxia and lactate in A549 VOC production, RNAseq was applied for further investigation to pursue A549-specific metabolic implications. We therefore focused on the VOC synthetic pathway, glucose and lactate metabolism (i.e., glycolysis) as well as the lactate signaling pathway (Figure 4; Supplementary Figure S1A).

VOC synthesis involves lipoperoxidation of PUFA. This consists of desaturation by desaturase, peroxidation by LOX and subsequent reduction of aldehydes by dehydrogenase (e.g., ADH). Desaturase tended to be downregulated in HBEpC under hypoxia, but to be upregulated in HLB under hypoxia. A549 tended to upregulate desaturase except under low glucose with 12.5 μM lactate addition. FAME analysis data showed no significant proportional increase of PUFFAs or PUFAMEs in any cell line; instead, some cases showed a decrease of PUFFAs and PUFAMEs (Supplementary Figure S5). The RNAseq data of desaturase apparently did not fit the cellular PUFAs and PUFAMEs data, which showed no increase (Supplementary Figure S5). One plausible explanation is that cellular PUFAs and PUFAMEs can be increased by desaturase upregulation in HLB and A549, but these reacted with ROS and were metabolized by upregulated LOXs.

LOXs are important genes for lipid peroxidation, which are mostly upregulated in all cell types. In particular, there was no downregulation in A549 (Figure 4). Regarding dehydrogenase or oxidoreductase enzymes (e.g., ADH), HBEpC and HLB downregulated most of them (including ADH) under hypoxia, whereas A549 rather upregulated them (Figure 4).

Regarding glucose metabolism, we compared the glucose transporter SLC2A families and rate-limited enzymes in glycolysis (e.g., HK, PFK, PKM) (Zuo et al., 2021). SLC2A genes were downregulated with lactate addition in A549, but no tendency was evident in HBEpC and HLB (Supplementary Figure S6). PFK showed no significant changes, and PKM (solely) showed simple upregulation (except HBEpC under 125 μM lactate addition). The HK-related gene (e.g., HK1, HK2) expression change does not appear to be related to lipid peroxidation (Supplementary Figure S6).

As for lactate metabolism, lactate transport is either from the cell to the outside or *vice versa*. MCT1 can play role in lactate uptake into cells, and MCT4 plays a role in the export from the cell to the outside (Ippoloto et al., 2019). However, no MCT showed a statistically significance change. The LDH-A type upregulated in all cell types under hypoxia, indicating that adding lactate into the medium enhances gene expression of LDH-A rather than suppressing it. Hence, the metabolic conversion of lactate into pyruvate was not directly linked with VOC production, suggesting a potential role of lactate in signal transduction.

There are several lactate signaling pathways, for example GPR- (G-protein coupled receptor) and NDRG-based signaling pathways. Lactate generated by cancer cells reportedly activates GPR81, leading to various functions such as angiogenesis and immune evasion (Brown and Ganapathy, 2020). In our RNAseq data, GPR81 did not show any statistically significant change in any cell type. Moreover, GPRs appeared to be mostly upregulated under low glycose *versus* high glucose conditions. NDRGs are also related to the lactate signaling pathway. Indeed, NsDRG1 was upregulated in all cell types under hypoxia. NDRG3, in turn, was downregulated in HBEpC but upregulated in HLB and most A549 cells under hypoxia (except A549 under low glucose with 12.5 μM lactate). Lactate can promote a hypoxic response independently of the HIF signal by binding to NDRG3 protein for stabilization. This mediates the hypoxia-induced activation of the Raf-ERK pathway, leading to cell growth (Lee et al., 2015). NDRG3 might be related to cell growth of HLB and A549 under hypoxia, as characteristics of proliferating cells. NDRG2 is thought to mediate antitumor activity (Kim et al., 2021), and it was changed only in A549 cells (Supplementary Figure S6). Interestingly, it was upregulated under high glucose hypoxia but downregulated under low glucose hypoxia. Other genes related to lactate signaling—e.g., PGC-1, POMC, AGRP, and UCP–were not present in statistically significant amounts.

## Conclusion

We previously showed that *Trans* 2-hexenol was extensively emitted by A549 cancer cells under normoxia and high glucose conditions (compared with HBEpC primary cells and HLB non-cancer immortalized cells). Consistent with our finding, a recent study has shown that 2-hexenol can be a biomarker in urine for human lung cancer patients (Gasparri et al., 2022). In this study, *Trans* 2-hexenol was generated from the conversion of *Trans* 2-hexenal by alcohol dehydrogenase. Based on a comparison between different culture media, the production of such VOCs in A549 cancer cells could be enhanced under hypoxia and by lactate addition. It is interesting that lipid peroxidation can be enhanced under hypoxia (i.e., low oxygen concentration), despite the decrease in oxygen. This implies an unknown connection between the hypoxia signaling pathway and the VOC pathway. At the same time, glutaminolysis seems to influence both growth and the VOC pathway.

The comparison between A549 cancer cells and HLB non-cancerous immortalized cells provided important insights. Although both cell types showed difference in VOC production, both are proliferating cells possessing common metabolic characteristics (i.e., glutaminolysis and the Warburg effect). An difference between HLB and A549, for instance, was observed in cellular ADH and related oxidoreductase expression under hypoxia as well as lactate signaling (i.e., NDRG2). In this study, we have not compared many other cell lines. Nonetheless, there is a report that states lipid peroxidation can be enhanced hypoxia and lactate shuttle itself has been reported as characteristics of cancer cell in several cell lines. As such, it is amenable to envisage that an effect of hypoxia and lactate to lipid peroxidation and VOC production might be common characteristics of cancer cells. Determining the details of the potential correlation between hypoxia, lactate signaling, and lipid peroxidation warrants further study in order to decipher VOC metabolism.

## Data Availability

The datasets presented in this study can be found in online repositories. The names of the repository/repositories and accession number(s) can be found in the article/Supplementary Material.

## Supplementary material

SUPPLEMENTARY FIGURE S1
**(A)**, Cellular metabolic pathway and signal transduction potentially related to VOC production; **(B)**, Ten different culture conditions used in this study. Variants are glucose concentration (high or low), glutamine (present or absent), Aerobic/Hypoxia, and addition of lactate (0–1.25 mM).

SUPPLEMENTARY FIGURE S2 Retention time and m/z of FFAs and FAMEs used for EIC quantification. C17:0 FFA and C17:0 FAME are internal standards. sFA, saturated fatty acid; mFA, monounsaturated fatty acid; puFA, polyunsaturated fatty acid; sFAME, saturated fatty acid methyl ester; mFAME, monounsaturated fatty acid methyl ester; puFAME, polyunsaturated fatty acid methyl ester. FA is free fatty acid and FAME is derived from bounded fatty acids. There are isomers in unsaturated C18, C20, C22 FAs, and FAMEs, which were chromatographically separated but showed an identical or very similar fragmentation pattern.

SUPPLEMENTARY FIGURE S3
**(A)**, Comparison of VOC production under different culture conditions (low glucose, hypoxia, lactate addition, glutamate deprivation). X axis is cell proliferation rate [Cell number (Day X / Day 0)] and Y axis is ΔVOC [trans-2-hexenol nmole (DayX-Day 0)]. Day X is either Day 2 or Day 3. Plots were statistically significant only compared to Day 0 (as shown in Figure 3A); **(B)**, Cellular (HBEpC, HLB and A549) LDH-B enzymatic assay. The comparison is between normal aerobic high glucose and any other culture condition. * statistically significant based on Holm–Bonferroni method (i.e., *p* < 0.0055). Color of * (red and blue) indicates either upregulated or downregulated.

SUPPLEMENTARY FIGURE S4 Glucose consumption measurement by Phenol-Sulfuric Acid Assay and medium lactate assay (negative control cell-free medium, HBEpC, HLB, and A549). The comparison group was divided into 2 groups (high glucose and low glucose medium). Glucose consumption was calculated based on Δ glucoses in the medium (Day 0-Day 3) / μL medium. Medium lactate was calculated as ng lactate / μL medium at Day 3. Statistical significance is based on the Holm–Bonferroni method (i.e., p<0.012). Color of * (red and blue) indicates either upregulated or downregulated.

SUPPLEMENTARY FIGURE S5 Cellular (HBEpC, HLB and A549) PUFFAs and PUFAMEs proportion in total lipid. Free fatty acids were derivatized by MSTFA and bounded fatty acids were methyl esterified. Comparison is between normal aerobic high glucose to all other culture conditions. Statistical significance is based on the Holm–Bonferroni method (i.e., *p* < 0.0055). Blue color * indicates downregulation.

SUPPLEMENTARY FIGURE S6 Mean fold value reflecting change in gene expression pattern based on RNAseq data (HBEpC, HLB and A549). Lipid peroxidation (VOC synthesis) pathway (DES, LOX, ADH) Glucose consumption and glycolysis (SLC2A, HK), and lactate metabolism and signaling (LDH, NDGR) are three main focuses. Six different culture conditions were compared, normoxia high glucose, hypoxia high glucose, hypoxia low glucose, and hypoxia low glucose with lactate (12.5 μM , 125 μM, or 1.25 mM). Only annotated genes showing statistical significance (q < 0.05) were displayed with either upregulated or downregulated compared to the control, which is the normoxia high glucose condition (Supplementary Material). HK, hexokinase families; SLC2A, solute carrier group of membrane transport proteins as glucose transporter; LDH, lactate dehydrogenase; NDGR, regulator for nitrogen source-dependent growth and antibiotic production.

## Abbreviations

A549, uman lung fibroblasts; ADH, alcohol dehydrogenase; DMEM, Dulbecco’s modified eagle medium; GC, gas chromatography; EI, electron ionization; FFAs, free fatty acids; FAMEs, fatty acid methyl esters; HBEpC, human bronchial epithelial cells (primary cell); HLB, non-cancer immortalized cell; MS, mass spectrometry; MSTFA, N-Methyl-N-trimethylsilyltrifluoroacetamide; PTR, Proton transfer reaction; PUFFAs, polyunsaturated free fatty acids; PUFAMEs, polyunsaturated fatty acid methyl esters; ROS, reactive oxygen species; SIFT, Selected ion flow tube; VEM-1, VOC Enrichment Machine; VOCs, volatile organic compounds.
