# Supplementary figures and images for: Hypoxia and lactate influence VOC production in A549 lung cancer cells
Source: Front Mol Biosci. 2023 Sep 21;10:1274298. doi: 10.3389/fmolb.2023.1274298 (PMC10552298; doi:10.3389/fmolb.2023.1274298)

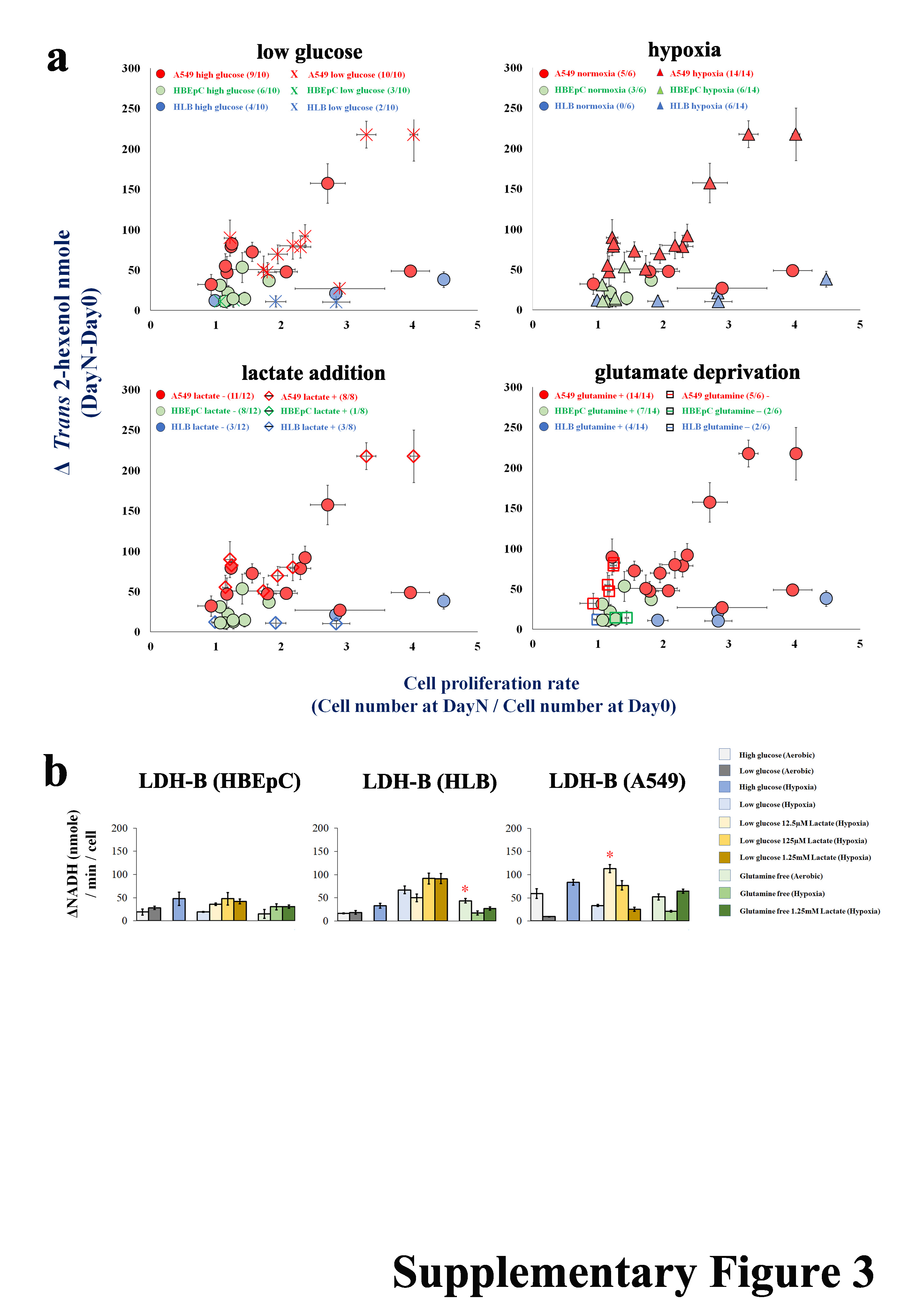

Supplement: Supplementary file 1 [file Image3.tiff]

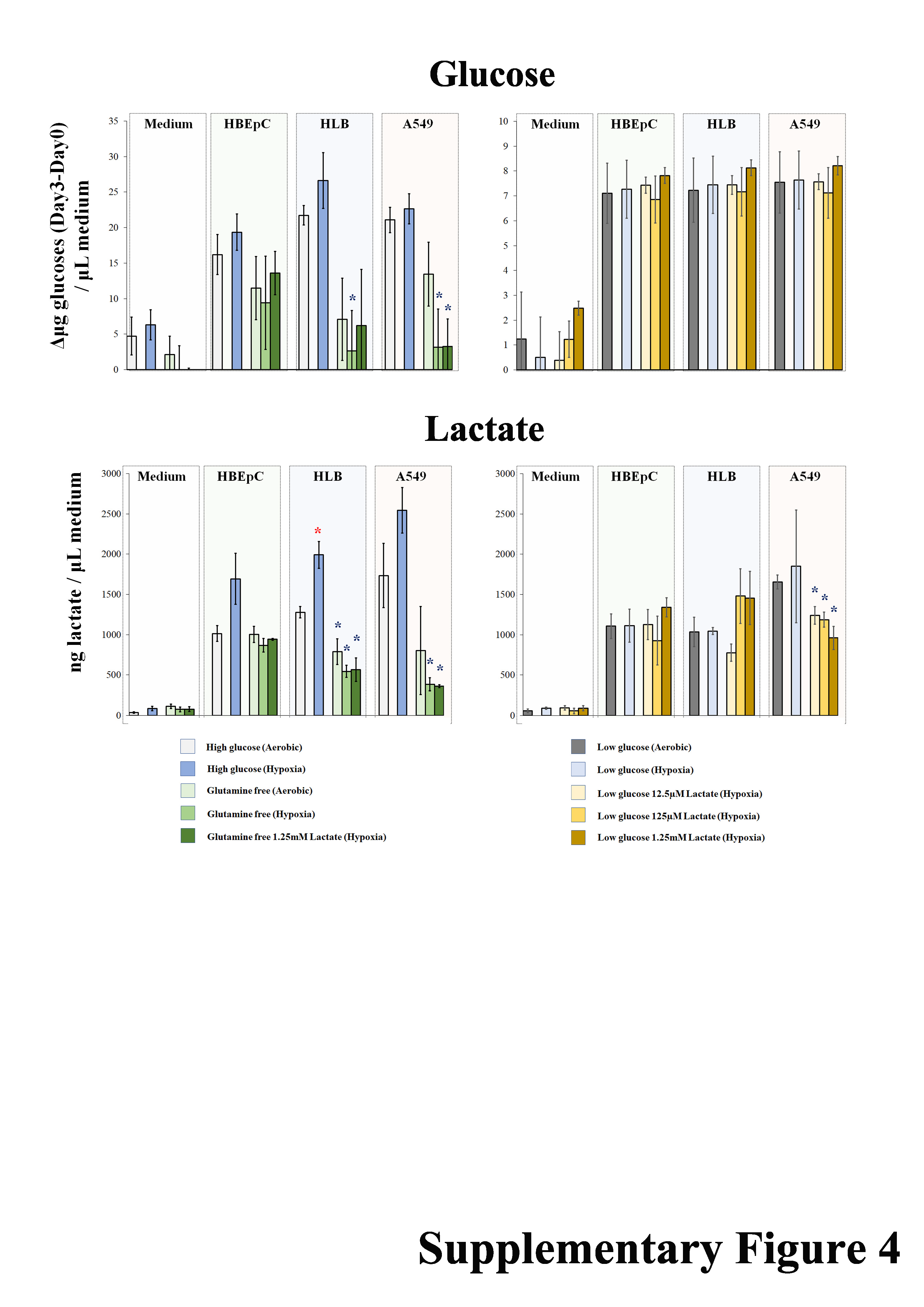

Supplement: Supplementary file 2 [file Image4.tif]

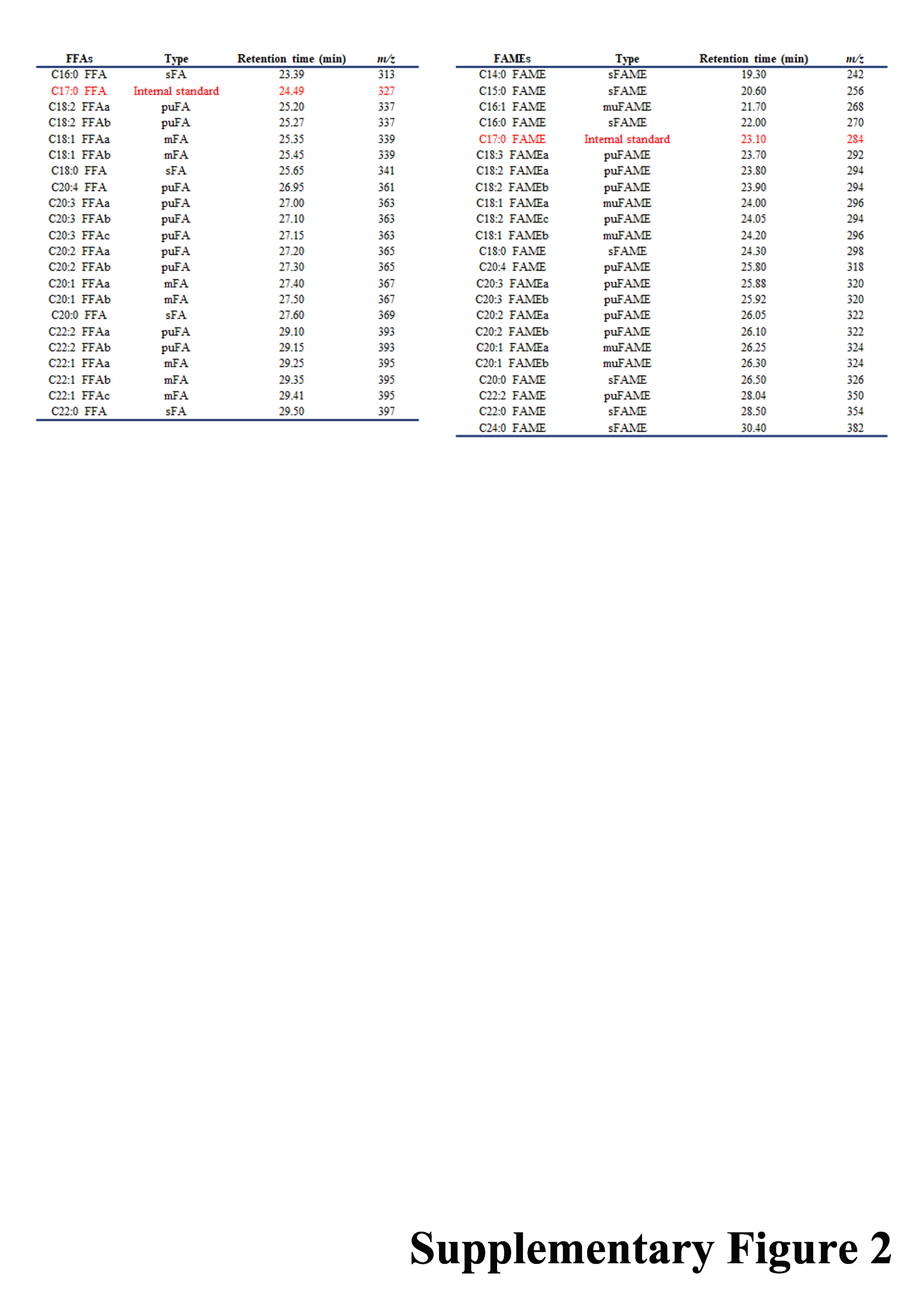

Supplement: Supplementary file 3 [file Image2.tif]

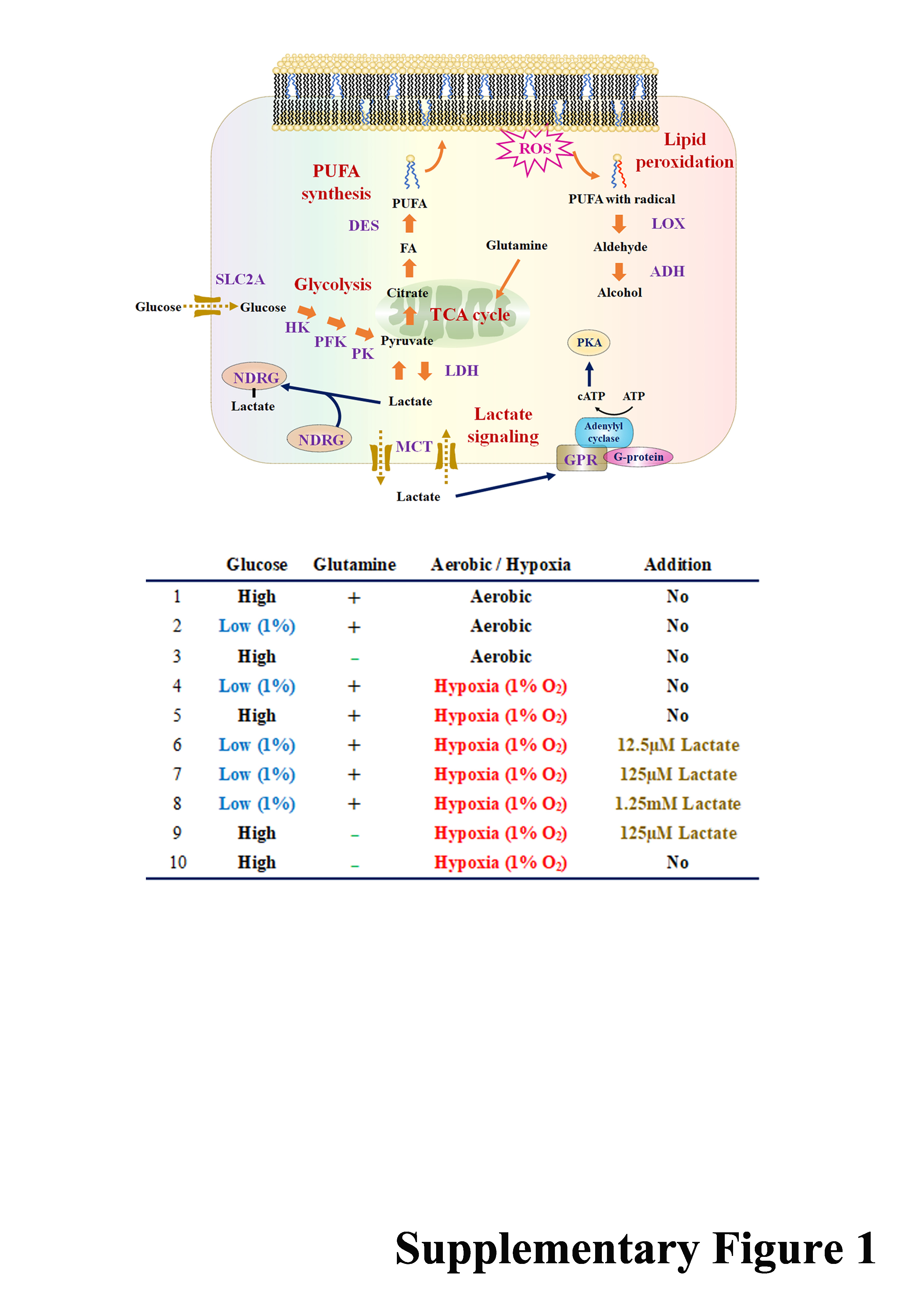

Supplement: Supplementary file 4 [file Image1.tif]

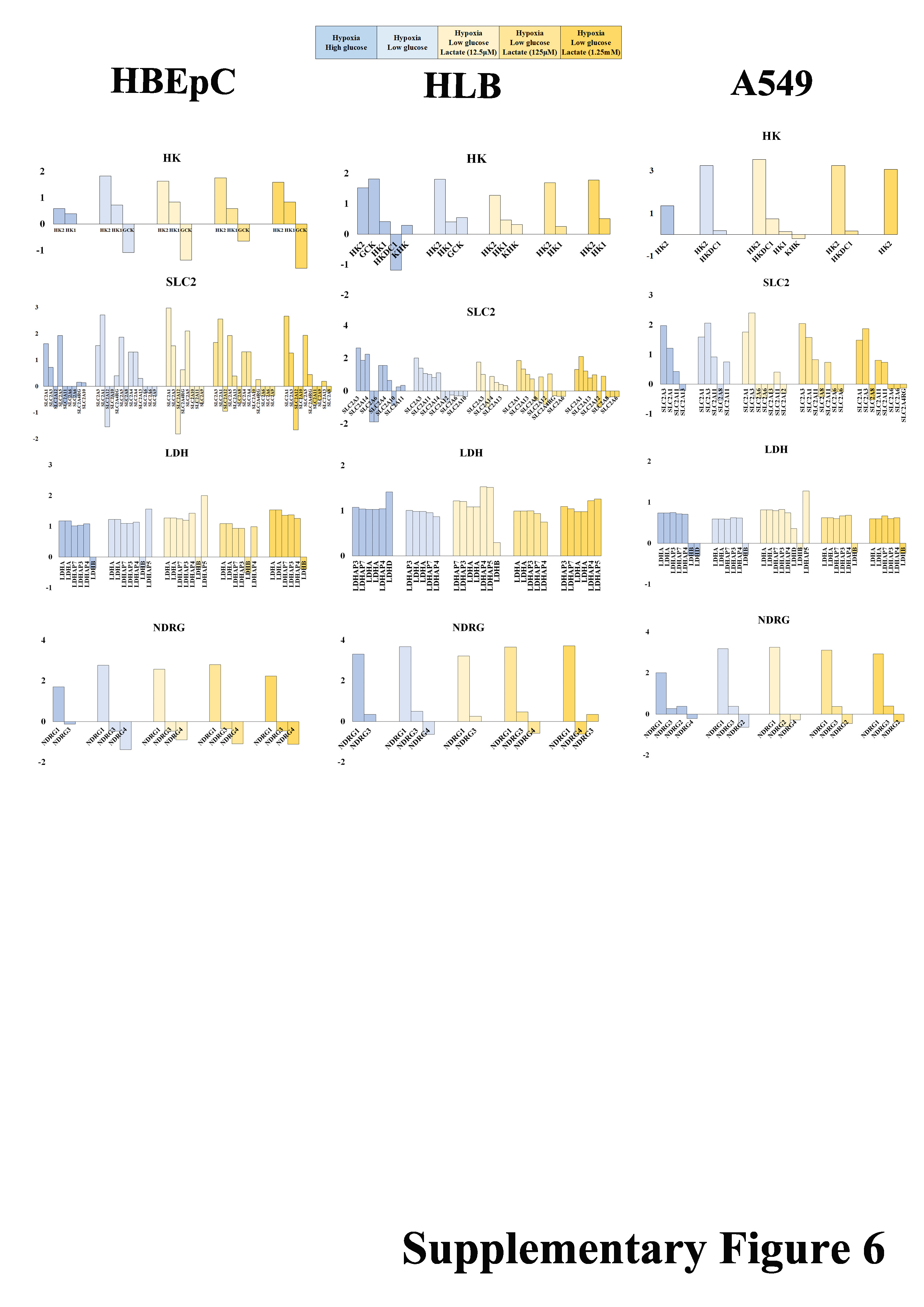

Supplement: Supplementary file 5 [file Image6.tiff]

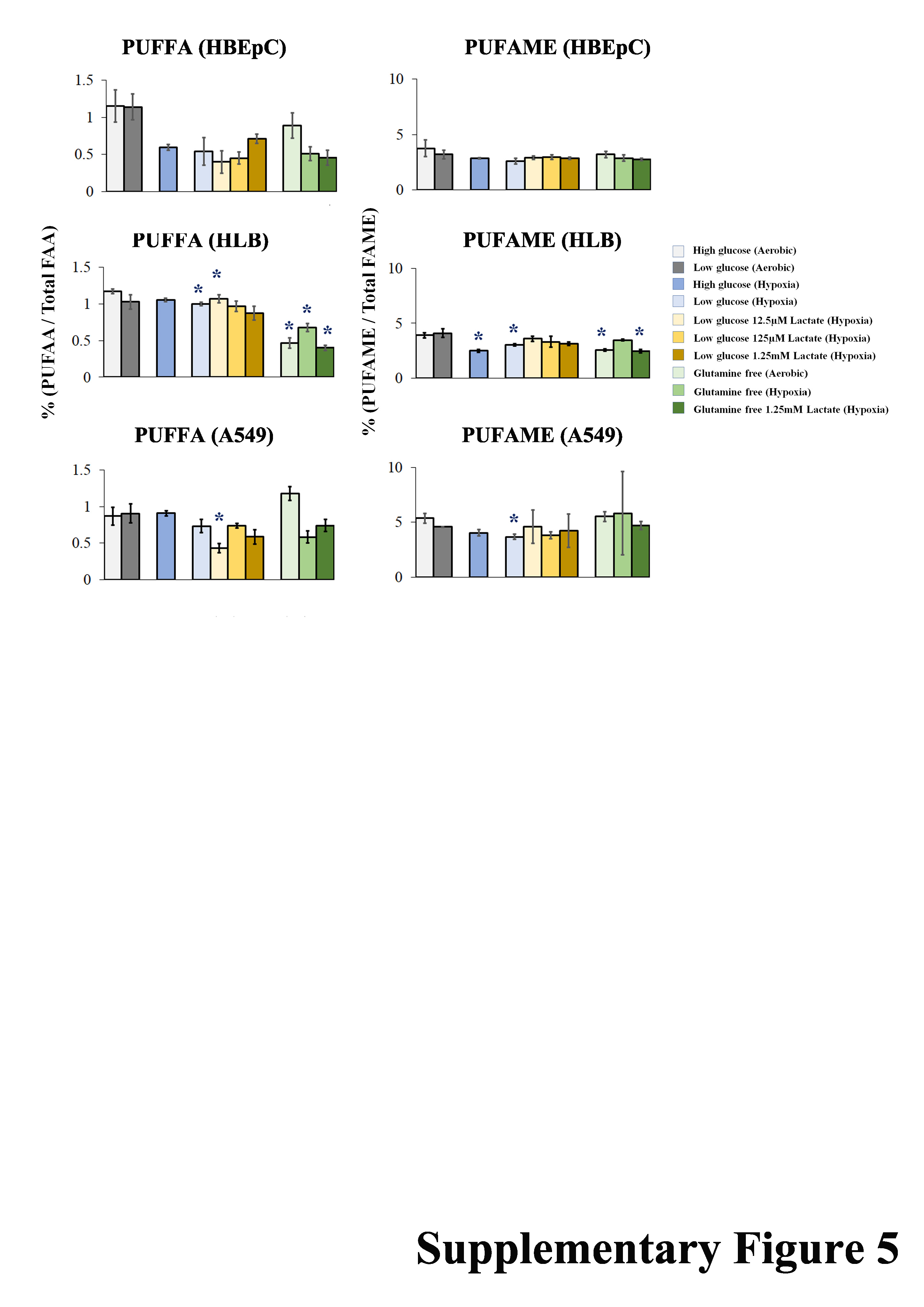

Supplement: Supplementary file 6 [file Image5.tif]
